# Supplementary material for: Pyruvate Dehydrogenase Kinase 1 inhibition mediated oxidative phosphorylation enhancement in cartilage promotes osteoarthritis progression
Source: BMC Musculoskelet Disord. 2023 Jul 20;24:597. doi: 10.1186/s12891-023-06585-6 (PMC10357736; doi:10.1186/s12891-023-06585-6)
Supplement: Supplementary file 1 — Additional file 1. [file 12891_2023_6585_MOESM1_ESM.zip › KEGG.Gsea.1653622432464/KEGG_CELL_CYCLE.html]

Details for gene set KEGG\_CELL\_CYCLE[GSEA]

|  || Dataset | 55457\_collapsed\_to\_symbols.cls.cls #OA\_versus\_normal.cls.cls #OA\_versus\_normal\_repos |
| Phenotype | cls.cls#OA\_versus\_normal\_repos |
| Upregulated in class | OA |
| GeneSet | KEGG\_CELL\_CYCLE |
| Enrichment Score (ES) | 0.17751303 |
| Normalized Enrichment Score (NES) | 0.8315197 |
| Nominal p-value | 0.82779455 |
| FDR q-value | 0.92223966 |
| FWER p-Value | 1.0 |
Table: GSEA Results Summary

  

Fig 1: Enrichment plot: KEGG\_CELL\_CYCLE      
 Profile of the Running ES Score & Positions of GeneSet Members on the Rank Ordered List

  

| SYMBOL | TITLE | RANK IN GENE LIST | RANK METRIC SCORE | RUNNING ES | CORE ENRICHMENT || 1 | YWHAH | tyrosine 3-monooxygenase/tryptophan 5-monooxygenase activation protein eta [Source:HGNC Symbol;Acc:HGNC:12853] | 118 | 0.889 | 0.0154 | Yes |
| 2 | SMAD2 | SMAD family member 2 [Source:HGNC Symbol;Acc:HGNC:6768] | 275 | 0.768 | 0.0245 | Yes |
| 3 | RBX1 | ring-box 1 [Source:HGNC Symbol;Acc:HGNC:9928] | 348 | 0.724 | 0.0389 | Yes |
| 4 | MCM5 | minichromosome maintenance complex component 5 [Source:HGNC Symbol;Acc:HGNC:6948] | 351 | 0.724 | 0.0587 | Yes |
| 5 | ORC5 | origin recognition complex subunit 5 [Source:HGNC Symbol;Acc:HGNC:8491] | 422 | 0.697 | 0.0725 | Yes |
| 6 | CDC20 | cell division cycle 20 [Source:HGNC Symbol;Acc:HGNC:1723] | 552 | 0.641 | 0.0802 | Yes |
| 7 | CDKN2A | cyclin dependent kinase inhibitor 2A [Source:HGNC Symbol;Acc:HGNC:1787] | 799 | 0.570 | 0.0767 | Yes |
| 8 | CDK7 | cyclin dependent kinase 7 [Source:HGNC Symbol;Acc:HGNC:1778] | 834 | 0.560 | 0.0895 | Yes |
| 9 | MCM2 | minichromosome maintenance complex component 2 [Source:HGNC Symbol;Acc:HGNC:6944] | 888 | 0.546 | 0.1005 | Yes |
| 10 | TFDP1 | transcription factor Dp-1 [Source:HGNC Symbol;Acc:HGNC:11749] | 1048 | 0.508 | 0.1021 | Yes |
| 11 | ANAPC13 | anaphase promoting complex subunit 13 [Source:HGNC Symbol;Acc:HGNC:24540] | 1116 | 0.493 | 0.1105 | Yes |
| 12 | MCM6 | minichromosome maintenance complex component 6 [Source:HGNC Symbol;Acc:HGNC:6949] | 1131 | 0.490 | 0.1230 | Yes |
| 13 | ORC3 | origin recognition complex subunit 3 [Source:HGNC Symbol;Acc:HGNC:8489] | 1148 | 0.487 | 0.1352 | Yes |
| 14 | ORC4 | origin recognition complex subunit 4 [Source:HGNC Symbol;Acc:HGNC:8490] | 1234 | 0.470 | 0.1416 | Yes |
| 15 | PTTG1 | "PTTG1 regulator of sister chromatid separation, securin [Source:HGNC Symbol;Acc:HGNC:9690]" | 1329 | 0.453 | 0.1468 | Yes |
| 16 | CDC6 | cell division cycle 6 [Source:HGNC Symbol;Acc:HGNC:1744] | 1341 | 0.451 | 0.1584 | Yes |
| 17 | CDK4 | cyclin dependent kinase 4 [Source:HGNC Symbol;Acc:HGNC:1773] | 1375 | 0.447 | 0.1682 | Yes |
| 18 | MAD2L1 | mitotic arrest deficient 2 like 1 [Source:HGNC Symbol;Acc:HGNC:6763] | 1516 | 0.427 | 0.1690 | Yes |
| 19 | PCNA | proliferating cell nuclear antigen [Source:HGNC Symbol;Acc:HGNC:8729] | 1557 | 0.419 | 0.1775 | Yes |
| 20 | MAD1L1 | mitotic arrest deficient 1 like 1 [Source:HGNC Symbol;Acc:HGNC:6762] | 2266 | 0.330 | 0.1313 | No |
| 21 | CCNB1 | cyclin B1 [Source:HGNC Symbol;Acc:HGNC:1579] | 2359 | 0.319 | 0.1329 | No |
| 22 | BUB1 | BUB1 mitotic checkpoint serine/threonine kinase [Source:HGNC Symbol;Acc:HGNC:1148] | 2388 | 0.315 | 0.1395 | No |
| 23 | CCNE2 | cyclin E2 [Source:HGNC Symbol;Acc:HGNC:1590] | 2469 | 0.306 | 0.1417 | No |
| 24 | TGFB1 | transforming growth factor beta 1 [Source:HGNC Symbol;Acc:HGNC:11766] | 2490 | 0.303 | 0.1485 | No |
| 25 | CCND2 | cyclin D2 [Source:HGNC Symbol;Acc:HGNC:1583] | 2508 | 0.302 | 0.1556 | No |
| 26 | ORC1 | origin recognition complex subunit 1 [Source:HGNC Symbol;Acc:HGNC:8487] | 2525 | 0.300 | 0.1626 | No |
| 27 | CHEK2 | checkpoint kinase 2 [Source:HGNC Symbol;Acc:HGNC:16627] | 2533 | 0.299 | 0.1703 | No |
| 28 | CDKN1B | cyclin dependent kinase inhibitor 1B [Source:HGNC Symbol;Acc:HGNC:1785] | 2984 | 0.251 | 0.1421 | No |
| 29 | ESPL1 | "extra spindle pole bodies like 1, separase [Source:HGNC Symbol;Acc:HGNC:16856]" | 3122 | 0.238 | 0.1380 | No |
| 30 | CHEK1 | checkpoint kinase 1 [Source:HGNC Symbol;Acc:HGNC:1925] | 3173 | 0.233 | 0.1405 | No |
| 31 | PLK1 | polo like kinase 1 [Source:HGNC Symbol;Acc:HGNC:9077] | 3556 | 0.198 | 0.1161 | No |
| 32 | BUB3 | BUB3 mitotic checkpoint protein [Source:HGNC Symbol;Acc:HGNC:1151] | 3656 | 0.190 | 0.1136 | No |
| 33 | MCM3 | minichromosome maintenance complex component 3 [Source:HGNC Symbol;Acc:HGNC:6945] | 3675 | 0.188 | 0.1174 | No |
| 34 | CDC23 | cell division cycle 23 [Source:HGNC Symbol;Acc:HGNC:1724] | 3729 | 0.183 | 0.1183 | No |
| 35 | CDK1 | cyclin dependent kinase 1 [Source:HGNC Symbol;Acc:HGNC:1722] | 3744 | 0.182 | 0.1223 | No |
| 36 | CDKN2C | cyclin dependent kinase inhibitor 2C [Source:HGNC Symbol;Acc:HGNC:1789] | 4016 | 0.155 | 0.1053 | No |
| 37 | SMAD3 | SMAD family member 3 [Source:HGNC Symbol;Acc:HGNC:6769] | 4061 | 0.150 | 0.1060 | No |
| 38 | GADD45A | growth arrest and DNA damage inducible alpha [Source:HGNC Symbol;Acc:HGNC:4095] | 4081 | 0.148 | 0.1087 | No |
| 39 | CCNH | cyclin H [Source:HGNC Symbol;Acc:HGNC:1594] | 4120 | 0.144 | 0.1097 | No |
| 40 | CDKN1C | cyclin dependent kinase inhibitor 1C [Source:HGNC Symbol;Acc:HGNC:1786] | 4233 | 0.134 | 0.1046 | No |
| 41 | CCND3 | cyclin D3 [Source:HGNC Symbol;Acc:HGNC:1585] | 4426 | 0.119 | 0.0929 | No |
| 42 | BUB1B | BUB1 mitotic checkpoint serine/threonine kinase B [Source:HGNC Symbol;Acc:HGNC:1149] | 4454 | 0.117 | 0.0940 | No |
| 43 | E2F5 | E2F transcription factor 5 [Source:HGNC Symbol;Acc:HGNC:3119] | 4542 | 0.110 | 0.0903 | No |
| 44 | CDC16 | cell division cycle 16 [Source:HGNC Symbol;Acc:HGNC:1720] | 4566 | 0.108 | 0.0914 | No |
| 45 | PTTG2 | pituitary tumor-transforming 2 [Source:HGNC Symbol;Acc:HGNC:9691] | 4621 | 0.103 | 0.0901 | No |
| 46 | STAG2 | stromal antigen 2 [Source:HGNC Symbol;Acc:HGNC:11355] | 4684 | 0.098 | 0.0879 | No |
| 47 | SMC1A | structural maintenance of chromosomes 1A [Source:HGNC Symbol;Acc:HGNC:11111] | 4942 | 0.077 | 0.0699 | No |
| 48 | STAG1 | stromal antigen 1 [Source:HGNC Symbol;Acc:HGNC:11354] | 5022 | 0.071 | 0.0657 | No |
| 49 | CDC14B | cell division cycle 14B [Source:HGNC Symbol;Acc:HGNC:1719] | 5099 | 0.064 | 0.0616 | No |
| 50 | E2F1 | E2F transcription factor 1 [Source:HGNC Symbol;Acc:HGNC:3113] | 5137 | 0.061 | 0.0604 | No |
| 51 | TFDP2 | transcription factor Dp-2 [Source:HGNC Symbol;Acc:HGNC:11751] | 5394 | 0.041 | 0.0415 | No |
| 52 | ANAPC10 | anaphase promoting complex subunit 10 [Source:HGNC Symbol;Acc:HGNC:24077] | 5424 | 0.039 | 0.0403 | No |
| 53 | TP53 | tumor protein p53 [Source:HGNC Symbol;Acc:HGNC:11998] | 5511 | 0.032 | 0.0344 | No |
| 54 | CCNE1 | cyclin E1 [Source:HGNC Symbol;Acc:HGNC:1589] | 5663 | 0.018 | 0.0231 | No |
| 55 | CDC25A | cell division cycle 25A [Source:HGNC Symbol;Acc:HGNC:1725] | 5666 | 0.018 | 0.0234 | No |
| 56 | YWHAZ | tyrosine 3-monooxygenase/tryptophan 5-monooxygenase activation protein zeta [Source:HGNC Symbol;Acc:HGNC:12855] | 5770 | 0.010 | 0.0157 | No |
| 57 | MCM4 | minichromosome maintenance complex component 4 [Source:HGNC Symbol;Acc:HGNC:6947] | 6003 | -0.005 | -0.0024 | No |
| 58 | RB1 | RB transcriptional corepressor 1 [Source:HGNC Symbol;Acc:HGNC:9884] | 6013 | -0.006 | -0.0029 | No |
| 59 | ATM | ATM serine/threonine kinase [Source:HGNC Symbol;Acc:HGNC:795] | 6179 | -0.019 | -0.0153 | No |
| 60 | TGFB3 | transforming growth factor beta 3 [Source:HGNC Symbol;Acc:HGNC:11769] | 6293 | -0.027 | -0.0234 | No |
| 61 | TTK | TTK protein kinase [Source:HGNC Symbol;Acc:HGNC:12401] | 6670 | -0.054 | -0.0513 | No |
| 62 | ANAPC5 | anaphase promoting complex subunit 5 [Source:HGNC Symbol;Acc:HGNC:15713] | 6751 | -0.059 | -0.0559 | No |
| 63 | RBL2 | RB transcriptional corepressor like 2 [Source:HGNC Symbol;Acc:HGNC:9894] | 6764 | -0.060 | -0.0552 | No |
| 64 | ANAPC2 | anaphase promoting complex subunit 2 [Source:HGNC Symbol;Acc:HGNC:19989] | 6787 | -0.062 | -0.0552 | No |
| 65 | SMC3 | structural maintenance of chromosomes 3 [Source:HGNC Symbol;Acc:HGNC:2468] | 6950 | -0.076 | -0.0658 | No |
| 66 | TGFB2 | transforming growth factor beta 2 [Source:HGNC Symbol;Acc:HGNC:11768] | 6985 | -0.079 | -0.0663 | No |
| 67 | MDM2 | MDM2 proto-oncogene [Source:HGNC Symbol;Acc:HGNC:6973] | 7273 | -0.101 | -0.0859 | No |
| 68 | CCND1 | cyclin D1 [Source:HGNC Symbol;Acc:HGNC:1582] | 7512 | -0.119 | -0.1012 | No |
| 69 | RBL1 | RB transcriptional corepressor like 1 [Source:HGNC Symbol;Acc:HGNC:9893] | 7776 | -0.140 | -0.1179 | No |
| 70 | HDAC1 | histone deacetylase 1 [Source:HGNC Symbol;Acc:HGNC:4852] | 7911 | -0.148 | -0.1243 | No |
| 71 | CCNA2 | cyclin A2 [Source:HGNC Symbol;Acc:HGNC:1578] | 8047 | -0.159 | -0.1305 | No |
| 72 | E2F2 | E2F transcription factor 2 [Source:HGNC Symbol;Acc:HGNC:3114] | 8090 | -0.162 | -0.1293 | No |
| 73 | SFN | stratifin [Source:HGNC Symbol;Acc:HGNC:10773] | 8118 | -0.164 | -0.1268 | No |
| 74 | CDK6 | cyclin dependent kinase 6 [Source:HGNC Symbol;Acc:HGNC:1777] | 8393 | -0.185 | -0.1431 | No |
| 75 | E2F4 | E2F transcription factor 4 [Source:HGNC Symbol;Acc:HGNC:3118] | 8445 | -0.190 | -0.1419 | No |
| 76 | SKP1 | S-phase kinase associated protein 1 [Source:HGNC Symbol;Acc:HGNC:10899] | 8454 | -0.190 | -0.1372 | No |
| 77 | CCNA1 | cyclin A1 [Source:HGNC Symbol;Acc:HGNC:1577] | 8584 | -0.200 | -0.1418 | No |
| 78 | SKP2 | S-phase kinase associated protein 2 [Source:HGNC Symbol;Acc:HGNC:10901] | 8625 | -0.203 | -0.1393 | No |
| 79 | GADD45G | growth arrest and DNA damage inducible gamma [Source:HGNC Symbol;Acc:HGNC:4097] | 8671 | -0.206 | -0.1371 | No |
| 80 | YWHAQ | tyrosine 3-monooxygenase/tryptophan 5-monooxygenase activation protein theta [Source:HGNC Symbol;Acc:HGNC:12854] | 8675 | -0.207 | -0.1317 | No |
| 81 | WEE1 | WEE1 G2 checkpoint kinase [Source:HGNC Symbol;Acc:HGNC:12761] | 8937 | -0.227 | -0.1458 | No |
| 82 | CDC27 | cell division cycle 27 [Source:HGNC Symbol;Acc:HGNC:1728] | 8944 | -0.227 | -0.1400 | No |
| 83 | SMAD4 | SMAD family member 4 [Source:HGNC Symbol;Acc:HGNC:6770] | 9035 | -0.235 | -0.1405 | No |
| 84 | ABL1 | "ABL proto-oncogene 1, non-receptor tyrosine kinase [Source:HGNC Symbol;Acc:HGNC:76]" | 9081 | -0.238 | -0.1374 | No |
| 85 | HDAC2 | histone deacetylase 2 [Source:HGNC Symbol;Acc:HGNC:4853] | 9359 | -0.260 | -0.1519 | No |
| 86 | CDC25C | cell division cycle 25C [Source:HGNC Symbol;Acc:HGNC:1727] | 9501 | -0.274 | -0.1554 | No |
| 87 | ANAPC1 | anaphase promoting complex subunit 1 [Source:HGNC Symbol;Acc:HGNC:19988] | 9630 | -0.286 | -0.1575 | No |
| 88 | PRKDC | "protein kinase, DNA-activated, catalytic subunit [Source:HGNC Symbol;Acc:HGNC:9413]" | 9632 | -0.286 | -0.1496 | No |
| 89 | CUL1 | cullin 1 [Source:HGNC Symbol;Acc:HGNC:2551] | 9762 | -0.297 | -0.1515 | No |
| 90 | CDC7 | cell division cycle 7 [Source:HGNC Symbol;Acc:HGNC:1745] | 9780 | -0.299 | -0.1445 | No |
| 91 | CDC45 | cell division cycle 45 [Source:HGNC Symbol;Acc:HGNC:1739] | 9982 | -0.319 | -0.1514 | No |
| 92 | FZR1 | fizzy and cell division cycle 20 related 1 [Source:HGNC Symbol;Acc:HGNC:24824] | 9996 | -0.321 | -0.1436 | No |
| 93 | MCM7 | minichromosome maintenance complex component 7 [Source:HGNC Symbol;Acc:HGNC:6950] | 10032 | -0.325 | -0.1373 | No |
| 94 | ORC2 | origin recognition complex subunit 2 [Source:HGNC Symbol;Acc:HGNC:8488] | 10191 | -0.340 | -0.1403 | No |
| 95 | CDC25B | cell division cycle 25B [Source:HGNC Symbol;Acc:HGNC:1726] | 10206 | -0.341 | -0.1319 | No |
| 96 | CDKN2B | cyclin dependent kinase inhibitor 2B [Source:HGNC Symbol;Acc:HGNC:1788] | 10324 | -0.353 | -0.1313 | No |
| 97 | RAD21 | RAD21 cohesin complex component [Source:HGNC Symbol;Acc:HGNC:9811] | 10510 | -0.374 | -0.1354 | No |
| 98 | CCNB2 | cyclin B2 [Source:HGNC Symbol;Acc:HGNC:1580] | 10639 | -0.387 | -0.1347 | No |
| 99 | CREBBP | CREB binding protein [Source:HGNC Symbol;Acc:HGNC:2348] | 10881 | -0.414 | -0.1421 | No |
| 100 | ZBTB17 | zinc finger and BTB domain containing 17 [Source:HGNC Symbol;Acc:HGNC:12936] | 10996 | -0.429 | -0.1392 | No |
| 101 | CDK2 | cyclin dependent kinase 2 [Source:HGNC Symbol;Acc:HGNC:1771] | 11069 | -0.441 | -0.1326 | No |
| 102 | GSK3B | glycogen synthase kinase 3 beta [Source:HGNC Symbol;Acc:HGNC:4617] | 11157 | -0.451 | -0.1269 | No |
| 103 | ATR | ATR serine/threonine kinase [Source:HGNC Symbol;Acc:HGNC:882] | 11231 | -0.461 | -0.1199 | No |
| 104 | PKMYT1 | "protein kinase, membrane associated tyrosine/threonine 1 [Source:HGNC Symbol;Acc:HGNC:29650]" | 11403 | -0.487 | -0.1197 | No |
| 105 | YWHAB | tyrosine 3-monooxygenase/tryptophan 5-monooxygenase activation protein beta [Source:HGNC Symbol;Acc:HGNC:12849] | 11488 | -0.500 | -0.1125 | No |
| 106 | ORC6 | origin recognition complex subunit 6 [Source:HGNC Symbol;Acc:HGNC:17151] | 11680 | -0.529 | -0.1128 | No |
| 107 | EP300 | E1A binding protein p300 [Source:HGNC Symbol;Acc:HGNC:3373] | 11741 | -0.540 | -0.1025 | No |
| 108 | DBF4 | DBF4 zinc finger [Source:HGNC Symbol;Acc:HGNC:17364] | 11855 | -0.561 | -0.0959 | No |
| 109 | YWHAE | tyrosine 3-monooxygenase/tryptophan 5-monooxygenase activation protein epsilon [Source:HGNC Symbol;Acc:HGNC:12851] | 12137 | -0.628 | -0.1005 | No |
| 110 | CDKN2D | cyclin dependent kinase inhibitor 2D [Source:HGNC Symbol;Acc:HGNC:1790] | 12296 | -0.676 | -0.0941 | No |
| 111 | CDKN1A | cyclin dependent kinase inhibitor 1A [Source:HGNC Symbol;Acc:HGNC:1784] | 12727 | -0.890 | -0.1031 | No |
| 112 | MYC | "MYC proto-oncogene, bHLH transcription factor [Source:HGNC Symbol;Acc:HGNC:7553]" | 12801 | -0.970 | -0.0820 | No |
| 113 | GADD45B | growth arrest and DNA damage inducible beta [Source:HGNC Symbol;Acc:HGNC:4096] | 12816 | -1.001 | -0.0554 | No |
| 114 | CDC14A | cell division cycle 14A [Source:HGNC Symbol;Acc:HGNC:1718] | 12823 | -1.009 | -0.0279 | No |
| 115 | E2F3 | E2F transcription factor 3 [Source:HGNC Symbol;Acc:HGNC:3115] | 12886 | -1.224 | 0.0011 | No |
Table: GSEA details [plain text format]

  

Fig 2: KEGG\_CELL\_CYCLE      
 Blue-Pink O' Gram in the Space of the Analyzed GeneSet

  

Fig 3: KEGG\_CELL\_CYCLE: Random ES distribution      
 Gene set null distribution of ES for **KEGG\_CELL\_CYCLE**
